# Algorithms for Finding Small Attractors in Boolean Networks

**DOI:** 10.1155/2007/20180

**Published:** 2007-04-12

**Authors:** Shu-Qin Zhang, Morihiro Hayashida, Tatsuya Akutsu, Wai-Ki Ching, Michael K Ng

**Affiliations:** 1 Advanced Modeling and Applied Computing Laboratory, Department of Mathematics, The University of Hong Kong, Pokfulam Road, Hong Kong; 2 Bioinformatics Center, Institute for Chemical Research, Kyoto University, Uji, Kyoto 611-0011, Japan; 3 Department of Mathematics, Hong Kong Baptist University, Kowloon Tong, Hong Kong

## Abstract

A Boolean network is a model used to study the interactions between different genes in genetic regulatory networks. In this paper, we present several algorithms using gene ordering and feedback vertex sets to identify singleton attractors and small attractors in Boolean networks. We analyze the average case time complexities of some of the proposed algorithms. For instance, it is shown that the outdegree-based ordering algorithm for finding singleton attractors works in  time for , which is much faster than the naive  time algorithm, where  is the number of genes and  is the maximum indegree. We performed extensive computational experiments on these algorithms, which resulted in good agreement with theoretical results. In contrast, we give a simple and complete proof for showing that finding an attractor with the shortest period is NP-hard.

## 

[1,2,3,4,5,6,7,8,9,10,11,12,13,14,15,16,17,18,19,20,21,22,23,24,25,26,27,28,29,30,31,32]
